# Perceived epilepsy-related stigma is linked to the socioeconomic status of the residence

**DOI:** 10.3389/fpubh.2022.952585

**Published:** 2022-08-26

**Authors:** Louisa Hohmann, Justus Berger, Shirley-Uloma Kastell, Martin Holtkamp

**Affiliations:** ^1^Epilepsy-Center Berlin-Brandenburg, Institute for Diagnostics of Epilepsy, Evangelisches Krankenhaus Königin Elisabeth Herzberge, Berlin, Germany; ^2^Epilepsy-Center Berlin-Brandenburg, Department of Neurology, Charité–Universitätsmedizin Berlin, Berlin, Germany

**Keywords:** seizures, neuropsychology, social deprivation, neurourbanism, social disadvantage, discrimination, structural socioeconomic status

## Abstract

**Purpose:**

Epilepsy is one of the most common neurological disorders with high costs for the healthcare systems and great suffering for patients. Beyond seizures, psychosocial comorbidities can have detrimental effects on the well-being of people with epilepsy. One source of social stress and reduced quality of life is epilepsy-related stigma that often occurs, e.g., due to public misconceptions or myths. Stigma has individual biological, psychological and social correlates. Moreover, environmental factors like living in remote areas are associated with stigma. However, little is known about the link between the social structure of the residence and stigma in epilepsy. Thus, we investigated the association between the structural socioeconomic status (SES) and perceived stigma in an urban epilepsy population.

**Methods:**

This prospective, cross-sectional study examined 226 adult in-patients with epilepsy from Berlin. Multiple regression analyses were performed to check the relationship between structural SES and stigma controlling for individual-level demographic, clinical, psychological and social factors. Continuous social indices (SI) of the districts and neighborhoods (“SI district” and “SI neighborhood”) of Berlin were used to measure different levels of structural SES. Non-linear relationships are tested by grouping the SI in quartiles.

**Results:**

Both indicators of structural SES were independently linked to stigma (*p* = 0.002). For “SI district”, we identified a non-linear relationship with patients from the most deprived quartile feeling less stigmatized compared to those in the second (*p* < 0.001) or least deprived quartile (*p* = 0.009). Furthermore, more restrictions of daily life (*p* < 0.001), unfavorable income (*p* = 0.009) and seizure freedom in the past 6 months (*p* = 0.05) were related to increased stigma. A lower “SI neighborhood” was associated with higher stigma (*p* = 0.002).

**Conclusion:**

Strategies to reduce epilepsy-related stigma need to consider the sociostructural living environment on different regional levels. Unfavorable relations with the immediate living environment may be directly targeted in patient-centered interventions. Non-linear associations with the structural SES of broader regional levels should be considered in public education programs. Further research is needed to examine possible underlying mechanisms and gain insight into the generalizability of our findings to other populations.

## Introduction

Epilepsy is one of the most common neurological disorders and characterized by the predisposition for the (repeated) occurrence of epileptic seizures (1). Up to 7 per 1,000 people suffer from epilepsy, and in developing countries prevalence rates are estimated even higher (2). Epilepsy possesses a large burden on the individual patients, on the communities they are living in, and on their healthcare systems (3).

Stigma refers to the “co-occurrence of labeling, stereotyping, separation, status loss, and discrimination in a context in which power is exercised” (p. 813) (4) and represents a fundamental cause of health inequalities. Furthermore, stigma has large ecological costs due to its negative impact on employment, income, public views about resource allocation, and healthcare costs (5). It is a frequent concern of patients with mental, somatic, and neurological disorders and their caregivers (6). Especially people with epilepsy (PWE) may be confronted with a particularly severe stigma, as the chronic disorder is often accompanied by public misconceptions, myths and negative attitudes (6). Many PWE suffer from detrimental effects of stigma on their well-being, e.g., negative feelings or higher stress (7, 8). Stigma represents a major limitation of quality of life (QoL) for PWE, even beyond seizure-related factors or other psychosocial comorbidities (9). Up to 80% of PWE report feeling stigmatized (10), but research shows a great variability in stigma prevalence depending on the specific patient population. This underlines that it is necessary to understand correlates of stigma to identify risk populations, and to develop adequate intervention and prevention strategies.

Previous research highlights that various factors are associated with higher stigma in PWE, e.g., greater seizure severity, more antiseizure medications (ASM), more ASM adverse events, poorer QoL, as well as more depressive and anxiety symptoms (11, 12). Stigma is socially determined and occurs in many social situations. Thus, beyond the aforementioned clinical and psychological factors, stigma depends on various social characteristics of the individual and their communities. Research on stigma against various health conditions finds associations with different aspects of the socioeconomic status (SES). This concept can be subdivided in an individual SES, covering an individual's education, occupation and income, as well as a structural SES, including the social structure of an individual's living environment (13). Social aspects on both levels are linked to stigma: For instance, perceived weight stigma depends on income and social support (14). Cancer stigma is related to social constraints and income (15), or negative attitudes toward mental illnesses are more pronounced in socially deprived areas (16).

Compared to the general population, PWE have lower individual and structural SES: They are more often unemployed, have lower educational levels, or live more often in socially deprived areas (17, 18). Crucially, health of PWE is determined by social factors on both levels, as for instance access to epilepsy care, epilepsy knowledge, and outcomes of medial and surgical treatment are related to the SES. Stigma plays an important role in this framework (19). Regarding the individual SES, for instance, PWE with poorer financial conditions suffer from more stigma. Moreover, living in an environment of low structural SES may be a source of greater epilepsy-related stigma which is higher in rural compared to urban areas in African countries (12) or in public compared to private hospitals in the US (20). Public misconceptions and lack of epilepsy-related knowledge may be more pronounced in these regions and settings (11). Furthermore, worse access to treatment in socially deprived areas (21) may lead to greater reduction of daily abilities which in turn may increase stigma. However, quantitative investigations of associations between the structural SES and epilepsy-related stigma are still sparse. Results from Houston, US, suggest that stigma may not only differ between urban and rural areas but also according to structural SES differences within the same city (20).

Inhabitants of larger cities are exposed to particularly high levels of social stress. Possibly pathogenic stress may be caused or at least influenced by the co-occurrence of high population density and social isolation (22). In this framework, stigma is suggested to be part of a vicious circle: On the one hand, people with higher stress levels may be more vulnerable for stigmatized conditions. On the other hand, stigma may lead to more social isolation and elevated stress responses (4, 23). However, mechanisms of pathological urban stress are still not clear. They are addressed in the new field of neurourbanism that is connecting research in neuroscience, architecture, mental health, urban planning, and sociology. Taking this interdisciplinary perspective, the identification of possible regional differences of stigma within a city may help to identify different risk and resilience factors (22). The current study aimed to contribute to the field of neurourbanism by shedding a more detailed light on stigma correlates within an urban population. Our results on links between perceived stigma and the structural SES of adult in-patients with epilepsy in Berlin may give hints to mechanisms in other health conditions as well. We hypothesized that a lower structural SES is associated with higher perceived stigma, even after controlling for demographic, clinical, psychological, and social characteristics on the individual level.

## Materials and methods

### Sample

Our cohort consisted of 226 adults (≥ 18 years old) with epilepsy residing in Berlin and represents a subsample of a larger prospective project on determinants of QoL in adults with seizure disorders (epilepsy, syncopes, psychogenic non-epileptic seizures). Diagnoses were made of the basis of detailed history taking by experienced epileptologists and, if necessary, by ictal long-term video EEG-recordings. The participants were in-patients at the Epilepsy-Center Berlin-Brandenburg, a large tertiary hospital treating patients from all over Berlin, between 01/2018 and 12/2021. Of the sample, 31% (*n* = 69) underwent long-term video-EEG monitoring in preparation of a possible surgical intervention to remove the seizure focus. Firstly, medical records were screened to apply the following exclusion criteria: (1) legal guardianship; (2) physical conditions impairing the ability to fill out questionnaires; (3) poor German language comprehension; (4) low cognitive or intellectual abilities. In addition to that, senior epileptologists with neuropsychiatric expertise had evaluated all patients to ensure that no severe mental illnesses such as schizophrenia, dementia, or bipolar disorders were present that may have resulted in invalid answers on the questionnaires. Furthermore, patients were contacted by trained neuropsychologists to exclude those with cognitive disturbances.

This study is approved by the Institutional Review Board of Charité–Universitätsmedizin Berlin (EA4/208/17). Patients were informed about a possible study participation at the beginning of their hospital stay. After they had given informed written consent, they filled out self-report questionnaires on different psychological, social and epilepsy-related variables via tablets. In rare cases of patients' difficulties with technical understanding, paper-pencil versions were used. Additional demographic and clinical variables were obtained from medical charts and databases.

### Measurement

#### Stigma

Perceived epilepsy-related stigma was assessed with the subscale “stigma” of an adapted version of the German Performance, socio-demographic aspects, subjective evaluation (PESOS) questionnaire (24, 25). On six questions on five-point rating scales the participants indicated how they experience other people's reactions or feelings related to their epilepsy in the past 6 months (English translation of a sample item: “Are others withdrawing from you due to your epilepsy?,” a full ad hoc translation can be retrieved from the Supplementary material). Values on the single items were added and transformed so that the final stigma score ranges between 0 (lowest level of stigma) and 100 (highest level of stigma). May et al. (24) reported a good internal consistency (α = 0.88) in a sample of 196 adults and proofs of validity of the scale (24).

#### Clinical variables

“Seizure severity” was assessed using a German version of the Liverpool Seizure Severity Scale (LSSS), a questionnaire on seizure characteristics, consequences, impairment and controllability (26). For instance, patients are asked whether their seizure occur with loss of consciousness or whether they could suppress their seizures. Sum scores across 20 items were calculated with higher scores reflecting higher severity. Internal consistency (α > 0.7) and validity were proven in previous research (27).

“ASM adverse events” were measured using a German translation of the Liverpool Adverse Events Profile (LAEP) (28). The occurrence of 19 common adverse events in physical, somatic and psychological domains (e.g., dizziness, stomach problems or attentional deficits) in the past 4 weeks is rated on four-point scales. The sum across all items was calculated (range 19–76), whereby values ≥ 45 indicate substantial ASM adverse events (29). The LAEP shows a good internal consistency (α = 0.85) and was validated previously (30).

“Seizure frequency” was assessed with an adapted item of the PESOS (25). It originally consists of six categories ranging from “no seizures in the past 6 months” to “one seizure per day or more.” For a more detailed description of our study population, we further added two categories indicating whether seizure freedom was present in the past year or for more than 2 years.

With respect to the current ASM treatment, “ASM mono- vs. polytherapy” at the beginning of the in-patient stay was considered.

#### Psychological variables

QoL was measured using the German version of the Patient-Weighted Quality of Life in Epilepsy Questionnaire (QOLIE-31-P) (31). To ensure a broad assessment of patients' QoL, we used the “Overall” subscore. It consists of two ratings about the current QoL and the QoL in the past 4 weeks. Higher scores (range 0–100) reflect better QoL. This subscale shows acceptable internal consistency (α = 0.79) and has been validated previously (31).

A German version of the disorder-specific questionnaire Neurological Disorders Depression Inventory for Epilepsy (NDDI-E) was used to measure “depressive symptoms” (32). This rapid screening tool contains six questions about symptoms in the past 2 weeks that do not overlap with adverse ASM events or cognitive deficits and represents a standard tool in epilepsy research (33, 34). A cut-off of ≥ 14 points indicates clinically significant depressive symptoms. The German version has been validated in previous studies and shows an acceptable internal consistency (α = 0.83) (32).

“Anxiety symptoms” were assessed with the validated German version of the Generalized Anxiety Disorder 7-item (GAD-7) scale (35). This scale contains seven items on the most prominent features of generalized anxiety disorder, e.g., irritability, muscle tension, or restlessness. The scale has been validated in PWE and has a high internal consistency (α = 0.92). Sum scores of at least 6 points indicate significant anxiety symptoms in PWE (36).

“Restrictions of daily life” were measured with the corresponding subscale of the PESOS. The scale comprises 14 items about problems with independent living, mobility, partnership, leisure time, family, friends, and mental/physical health during the past 6 months. The mean score (range 0–100) reflects the degree of perceived restrictions in daily life with high values corresponding to high disadvantage. Reliability (α = 0.91) and validity has been proven in previous research (24).

#### Social variables

##### Structural socioeconomic status

**Figure 1:**
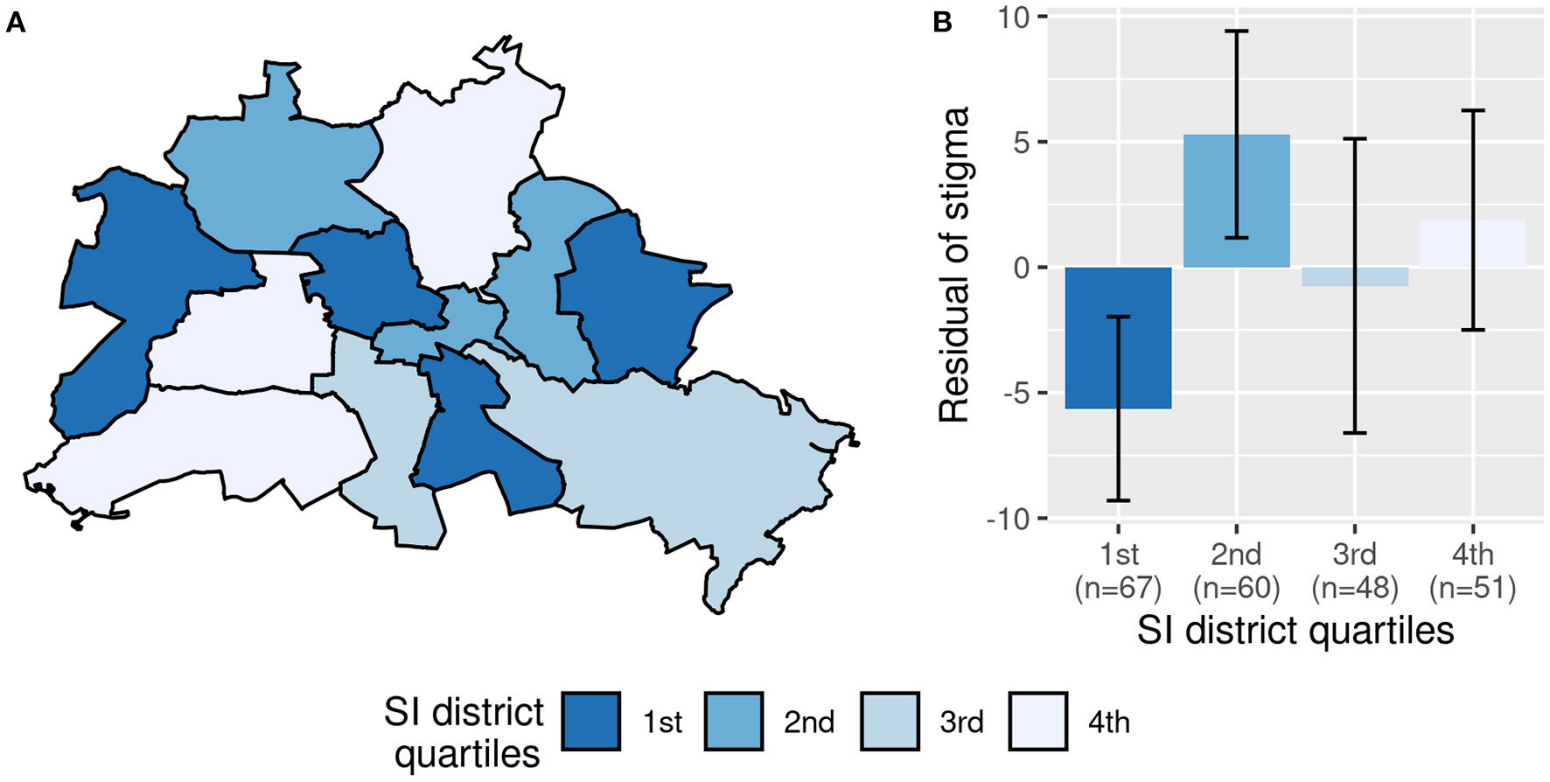
Association between perceived stigma and social index of the district (“SI district”). **(A)** shows Berlin with its 12 districts. **(B)** Displays the mean residual stigma values of the quartiles of SI district with error bars representing 95% confidence intervals around the mean. The residuals are corrected for social index of the neighborhood, restrictions of daily life, unfavorable income and seizures in the past 6 months.

**Figure 2:**
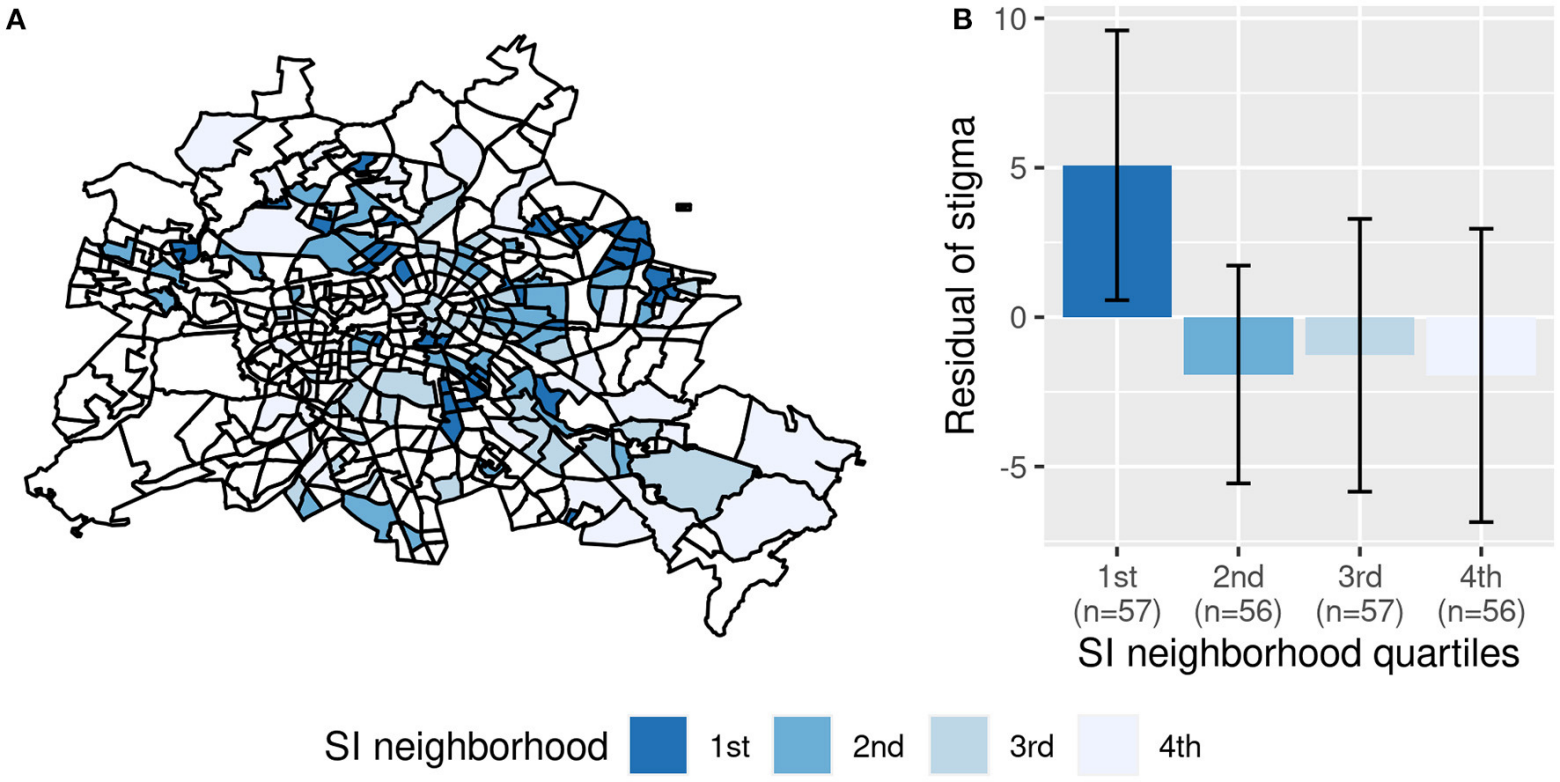
Association between perceived stigma and social index of the neighborhood (“SI neighborhood”). **(A)** Shows Berlin with its 447 neighborhoods. White areas did not contain any patients from our sample. **(B)** Displays the mean residual stigma values of the quartiles of SI neighborhood with error bars representing 95% confidence intervals around the mean. The residuals are corrected for social index of the district, restrictions of daily life, unfavorable income and seizures in the past 6 months.

The structural SES was measured with two differentiated social indices (SIs) of the social structure of Berlin summarizing various aspects of population, education, income, and health. These standardized scores (M = 0, SD = 1) are based on representative data of the city and are calculated for two different regional levels, 12 districts and 447 neighborhoods, by using factor analyses. Lower values for “SI district” and “SI neighborhood” indicate a lower structural SES, e.g., high unemployment rates, many inhabitants living on social welfare and low income, high premature and avoidable mortality, and many severe health problems due to tobacco abuse (37). Regional distributions are depicted in Figures 1A, 2A.

##### Individual social variables

Self-reports on education in the PESOS (25) were classified according to the revised International Standard Classification of Education (38). This classification system contains nine levels rating educational programs according to the degrees of complexity and specialization. Levels 0–2 were classified as “low education,” levels 3–4 were classified as “medium education” and levels 5–8 were classified as “high education.” The dichotomous variable on income indicated whether patients had an “unfavorable income,” i.e., if they received social welfare or reduced earning capacity.

### Statistical analysis

Data was analyzed with R Version 4.2.1 (39). Complete data on the relevant variables was available for all patients included in the study. We applied a multiple linear regression analysis to assess independent associations between the structural SES (“SI district,” “SI neighborhood”) and stigma. As possible confounders, variables identified as important correlates of stigma in the meta-analysis of Shi et al. (12) were considered. These comprise “unfavorable income,” “seizure severity,” “seizure frequency,” “number of ASM”, “ASM adverse events,” “depressive symptoms,” “anxiety symptoms,” and “quality of life.” “Seizure frequency” was initially measured using eight possible categories. For our analysis, we dichotomized the variable (“seizures in the past 6 months”, “no seizures in the past 6 months)” because including all eight categories would have resulted in small sample sizes for the distinct categories. Moreover, previous research shows that patient-related outcomes are not linearly associated with seizure frequency (40). Thus, by dividing the variable in two categories according to seizure freedom allows for a better theoretical interpretation than somewhat arbitrary cut-offs of the variable with respect to other categories.

In previous studies on German patient populations, stigma was associated with restrictions of everyday life (24). This variable was not considered in the meta-analysis that served as basis for the selection of additional predictors for stigma in our study (12). However, social support, a similar related construct, was identified as important correlate of stigma, and in a German publication that was not included in the meta-analysis, “restrictions of daily life” were strongly associated with stigma (11, 24, 25). We found a comparable spearman's rho correlation between “restrictions of daily life” and “stigma” (*r* = 0.59, *p* < 0.001). Therefore, we entered this variable in the multiple regression analysis. Stigma levels did not differ between patients undergoing presurgical assessment and other patients, *t*_(224)_ = −0.36, *p* = 0.72. Relevant predictors of stigma in the multiple regression analysis were selected based on the Akaike Information Criterion (AIC) with a forward-backward selection procedure using the function “stepAIC” (41). Multicollinearity was checked using variance inflation factors (VIFs) which were all in an appropriate range (VIF <10). Regression assumptions were checked graphically. According to the QQ-plot, normality of the residuals was violated in the first model. Therefore, influential data points according to Dffits-values were excluded and variable selection was again performed without these influential data points. The resulting model included the same predictors as the first model with similar *p*-values and slightly differing parameter estimates. Thus, this sensitivity analysis shows that the independent variables in the final model do not depend on extreme observations and can, therefore, be interpreted as important predictors of stigma. As a post hoc analysis, we checked non-linear relationships using quartiles of “SI district” and “SI neighborhood.” The analyses were reran with the confounders identified in the first multiple regression analysis. Differences between the quartiles were checked for statistical significance using a Bonferroni-corrected α-level of 0.008 for “SI district” and “SI neighborhood” separately. The models were compared using the AIC. Effect sizes were evaluated according to Cohen (42).

## Results

### Sample description

Demographic, clinical and social characteristics of the 226 patients are displayed in Table 1. In Table 2, answers on self-report questionnaires are presented. Compared to the general population of Berlin, our sample lived more frequently in socially deprived neighborhoods, *t*_(225)_ = −4.13, *p* < 0.001. For regional distributions, see Figures 1A, 2A.

**Table 1:** Sample description.

**Variable (*n* if not otherwise specified)**	**Value**
**Age** M ± SD, years	40.48 ± 15.49
Sex female	105 (46%)
Marital status (*n* = 224)	
single	140 (62%)
married	61 (27%)
other	23 (10%)
Unfavorable income: yes	74 (33%)
Employment	
employed	106 (47%)
in education	29 (13%)
unemployed	91 (40%)
Education	
low	37 (16%)
medium	110 (49%)
high	79 (35%)
Social index of district M ± SD	0.05 ± 0.89
Social index of neighborhood M ± SD	−0.25 ± 0.90
Age of epilepsy onset M ± SD, years	23.43 ± 17.10
Duration of epilepsy M ± SD, years	17.05 ± 14.54
Type of epilepsy	
focal	187 (82.7%)
generalized	23 (10.2%)
focal-generalized	1 (0.4%)
unclassified	15 (6.6%)
Possible epileptogenic lesions in MRI: yes	97 (43%)
Additional psychogenic non-epileptic seizures present: yes	12 (5%)
Additional syncopes present: yes	4 (2%)
Number of ASM M ± SD	1.47 ± 0.93
ASM polytherapy: yes	97 (43%)
Seizure frequency	
seizure free for 1 year to 2 years	13 (6%)
seizure free for 6 months to 1 year	8 (4%)
seizure free for the past 6 months	17 (8%)
1–2 seizures in the past 6 months	43 (19%)
3–5 seizures in the past 6 months	38 (17%)
1–2 seizures per month	49 (22%)
less than 1 seizure per day but at least 1 seizure per week	34 (15%)
at least 1 seizure per day	24 (11%)

ASM, antiseizure medications; MRI, magnet resonance imaging; SD, standard deviation. Diagnoses of epilepsy, psychogenic non-epileptic seizures, and syncopes were made of the basis of detailed history taking by experienced epileptologists and, if necessary, by ictal long-term video EEG-recordings.

**Table 2:** Answers on self-report questionnaires.

**Variable**	**M ±SD**	**Association with stigma**
Perceived stigma (PESOS)	24.69 ± 21.71	
Quality of Life (QOLIE-31-P)	58.38 ± 18.19	*r* = −0.39, *p* < 0.001^**^
Depressive symptoms (NDDI-E)	12.87 ± 3.97	*r* = 0.40, *p* < 0.001^**^
Anxiety symptoms (GAD-7)	8.00 ± 4.72	*r* = 0.42, *p* < 0.001^**^
Restrictions of daily life (PESOS)	32.43 ± 21.47	*r* = 0.59, *p* < 0.001^**^
Seizure severity (LSSS)	49.29 ± 8.49	*r* = 0.25, *p* = 0.001^*^
ASM adverse events (LAEP)	42.00 ± 10.74	*r* = 0.42, *p* < 0.001^**^

ASM, antiseizure medications; GAD-7, Generalized Anxiety Disorder Screener; LAEP, Liverpool Adverse Events Profile; LSSS, Liverpool Seizure Severity Scale; NDDI-E, Neurological Disorders Depression Inventory for Epilepsy; PESOS, Performance, socio-demographic aspects, subjective evaluation questionnaire; QOLIE-31-P, Quality of Life in Epilepsy Questionnaire.

* p < 0.01;

** p < 0.001.

### Associations between stigma and the structural SES

**Figure 3:**
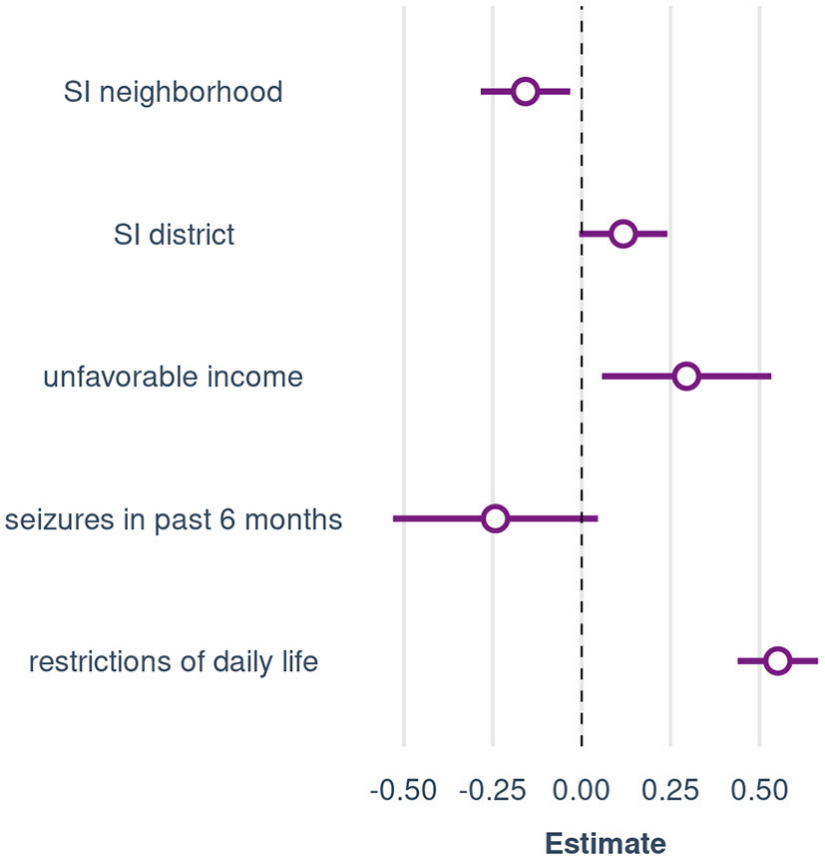
Variables independently associated with perceived stigma in the multiple regression analysis. Presented are regression coefficients and the 95% confidence intervals of the model with continuous values of SI neighborhood and SI district (Model 1). Coefficients are standardized for the continuous predictors. SI, social index.

In the univariate analysis, perceived stigma was associated with “SI neighborhood” (*r* = −0.15, *p* = 0.02) but not with “SI district” (*r* = 0.04, *p* = 0.52). The multiple regression analysis revealed that perceived stigma was independently associated with both indicators of structural SES, i.e., “SI district” and “SI neighborhood.” They explained a significant independent proportion of 1.8% of the variance of perceived stigma, corresponding to a weak to small effect, *f*^2^ = 0.03, 90%-CI [0.00, 0.07]. Furthermore, more restrictions of daily life, unfavorable income, and seizure freedom in the past 6 months were linked to higher perceived stigma (see Figure 3; Table 3, Model 1).

**Table 3:** Results of the multiple regression analysis.

	**Model 1 SI continuous**	**Model 2 SI categorized**
(Intercept)	25.8 (3.0, *p* < 0.001)	27.8 (3.7, *p* < 0.001)
SI neighborhood	−3.81 (1.54, p = 0.014)^*^	
SI district	2.86 (1.54, *p* = 0.064)	
SI neighborhood		
1^st^ vs. 2^nd^ quartile		−8.3 (3.3, *p* = 0.012)
1^st^ vs. 3^rd^ quartile		−9.3 (3.7, *p* = 0.011)
1^st^ vs. 4^th^ quartile		−10.4 (3.8, *p* = 0.007)^**^
2^nd^ vs. 3^rd^ quartile		−1.0 (3.3, *p* = 0.761)
2^nd^ vs. 4^th^ quartile		−2.1 (3.5, *p* = 0.551)
3^rd^ vs. 4^th^ quartile		−1.0 (3.2, *p* = 0.743)
SI district		
1^st^ vs. 2^nd^ quartile		11.9 (3.1, *p* < 0.001)^***^
1^st^ vs. 3^rd^ quartile		6.9 (3.6, *p* = 0.058)
1^st^ vs. 4^th^ quartile		9.9 (3.7, *p* = 0.009)^**^
2^nd^ vs. 3^rd^ quartile		−5.0 (3.5, *p* = 0.153)
2^nd^ vs. 4^th^ quartile		−2.0 (3.6, *p* = 0.579)
3^rd^ vs. 4^th^ quartile		3.0 (3.4, *p* = 0.378)
Unfavorable income	6.4 (2.6, *p* = 0.015)^*^	6.8 (2.6, *p* = 0.009)^**^
Seizures in the past	−5.3 (3.2, *p* = 0.099)	−6.2 (3.1, *p* = 0.048)^*^
6 months		
Restrictions of daily life	0.6 (0.06, *p* < 0.001)^***^	0.6 (0.06, *p* < 0.001)^***^
*R*^2^ / *R*^2^ adjusted	38.9% / 37.5%	42.8% / 40.37%
AIC	1290.77	1284.12
*f*^2^ [90%-CI	0.64 [0.43;0.84]	0.75 [0.50;0.95]
interval]		

Presented are unstandardized regression coefficients with standard errors and p-values in brackets. The intercept of Model 2 is calculated for the 1st quartiles of SI district and SI neighborhood as reference categories. The column contains all possible group comparisons for the quartiles. They were checked for statistical significance with a Bonferroni-corrected α-level of 0.008 and marked in the table accordingly. AIC, Akaike Information Criterion; SI, social index;

* p < 0.05;

** p < 0.01;

*** p < 0.001. CI, confidence interval.

Grouping both “SI district” and “SI neighborhood” into quartiles increased model fit, AIC = 1,284 vs. AIC = 1,290 of the continuous model, suggesting a non-linear relationship with at least one of the indicators. Together, the grouped variables of “SI district” and “SI neighborhood” explained a significant independent proportion of 5.6% of the variance in perceived stigma, corresponding to a small to medium effect, *f*^2^ = 0.10, 90%-CI [0.02,0.16]. Bonferroni-corrected post-hoc comparisons showed that perceived stigma was significantly higher in the most deprived neighborhood quartile compared to the least deprived neighborhood quartile (Figure 1B). Regarding the districts, perceived stigma was lower in the most deprived quartile compared to the second and fourth quartiles (see Table 3, Model 2; Figure 2B).

## Discussion

This study investigated the relationship between perceived stigma and the socioeconomic status of the residence (structural SES) in a sample of adult in-patients of a tertiary epilepsy center in Berlin. We found that even within one city, the structural SES on two different regional levels, i.e., immediate neighborhoods and more proximal districts, was associated with perceived stigma beyond individual-level demographic, clinical psychological, and social characteristics. Firstly, we investigated continuous measures of the structural SES, but, interestingly, our analyses showed that a categorization of the measures improved model fit. This finding indicates that the relationships between perceived stigma and structural SES follow non-linear trends. Thus, a conclusion “the higher the structural SES, the lower the stigma” is fairly too simple.

### Correlates of perceived stigma

#### Structural SES of the neighborhood

As expected, a lower structural SES of the neighborhood was associated with higher perceived stigma. Also after categorization, living in the most deprived neighborhood quartiles was linked to the highest levels of perceived stigma. This finding extends previous research in PWE showing that perceived stigma was higher in public compared to private hospitals in the US (20). Our indicator of structural SES not only covered the areas' healthcare systems but also includes broader indicators of population, income and health (37). Thus, one may conclude that higher social resources of different domains in the immediate living environment may be protective against stigma.

#### Structural SES of the district

Relationships differed for the broader regional SES level. In our continuous analyses, lower structural SES of the district was linked to lower stigma levels, which was contrary to our expectations. A closer examination of possible non-linear trends, the categorization in quartiles revealed that especially patients from the lowest SES districts reported fewer stigma than those from the second or least deprived districts. In PWE similar findings have not been identified previously. However, in women with human immunodeficiency viruses (HIV), living in areas with lower median income or more racial diversity was linked to lower perceived stigma (43). The authors concluded that a more diverse community may be protective against stigma. “SI district,” our measure of structural SES, does not include indicators of diversity. However, in districts of the lowest SI quartile, e.g. Berlin-Mitte or Neukölln, the rates of people with migrant background (55 or 49% in 2021) were highest. Moreover, the diversity of the countries of origins was largest, indicating greater cultural variety in these districts (44). Epilepsy-related stigma seems to be highly culture-specific (45). Our findings may suggest that living in areas with different cultures may bring different attitudes on epilepsy closer together, which may foster understanding and tolerance, and, therefore, result in lower stigma.

Moreover, previous studies found that mental health stigma was higher in individuals of higher SES (46). As an explanation of this finding, it was proposed that people with high SES experience more controllability in their life and more likely attribute causes of problems to controllable factors. This may lead to the assumptions that PWE may be more responsible for their symptoms and comorbidities and therefore increase negative attitudes and discriminating behaviors. Thus, this might have been another possible mechanism why perceived stigma was increased in higher SES districts in our study.

#### Other correlates

Perceived restrictions of everyday life were the most important predictor of stigma in our model. The (univariate) correlation was similar to that from a previously identified correlation (24). Our finding is also in line with theoretical models suggesting that problems in everyday social domains, e.g., with family and friends, leisure time activities or education and employment, may lead to feelings of higher stigma (19). However, causal conclusions cannot be drawn from our cross-sectional analysis. Therefore, the observed association may also represent effects in the opposite direction, i.e., detrimental effects of perceived stigma on social everyday functioning.

Moreover, patients who were seizure-free in the past 6 months reported more stigma. This finding is counterintuitive and somewhat unexpected as it contradicts previous research (11). In the public, seizures represent the key characteristic of epilepsy and are surrounded by many stigmatizing false beliefs (47). Thus, it may be reasonable that seizure freedom leads to less perceived stigma. However, our contrary finding may be due to the fact that seizure freedom does not mean that PWE may not suffer from other epilepsy-related problems, for instance cognitive problems, depressive symptoms or ASM adverse effects (48–50). All study participants were in-patients of the epilepsy center. Thus, also those who were seizure-free needed medical treatment due to conditions limiting their health. Possibly, these problems may be even greater sources of stigma than seizures themselves. Public stereotypes beyond seizures regarding PWE include that they are seen as over-anxious, antisocial, aggressive or retarded (47). Thus, PWE suffering from anxiety, behavioral or cognitive complaints may identify themselves stronger with these perceptions which may increase perceived stigma.

### Is the SES relevant for stigma?

In their systematic review, Baker et al. (45) summarized studies showing that the association between individual SES characteristics and stigma disappeared after controlling for psychological variables such as depression or QoL. Therefore, they concluded that the association between an individual person's SES and stigma rather reflects overlap between SES and other psychological variables. However, in contrast to this explanation, we found that individual as well as structural SES were more important in predicting perceived stigma than other demographic, clinical and psychological variables. We did not identify relevant multicollinearity of psychological and SES variables. Moreover, even after re-entering “QoL,” “depressive symptoms” and “anxiety symptoms” as possible predictors, stigma was still significantly associated with “SI district,” “SI neighborhood” and “unfavorable income.” Three of five predictors in the final model were related to the individual or structural SES, and they explained 7.7% of the variance of stigma. What is more, the variable “restrictions of daily life,” the most important predictor of stigma, represents a patient-rating of social everyday functioning. Thus, it may be seen as a social correlate in the broader sense. All in all, our findings indicate that social variables on different levels represent important correlates of perceived stigma and that they, at least in our sample, are even more important than psychological characteristics.

### Clinical consequences and stigma reduction strategies

Our finding of a negative association between “SI neighborhood” and perceived stigma may reflect that the immediate living environment of PWE in socially deprived neighborhoods offers fewer resources for social support or for developing coping abilities. In these areas, patient-based interventions to enhance social competencies, self-esteem, coping and epilepsy knowledge (51) may be less available for PWE and their caregivers. As epilepsy often leads to reduced mobility, especially in deprived areas, psychosocial and psychoeducational interventions should be installed.

In addition to individual interventions, public awareness interventions are relevant all over the city. For instance, lectures, entertainment events, or public service announcements, should be used to increase knowledge about epilepsy in the general population and also in PWE themselves (51). Our non-linear association between “SI district” and stigma leads to the conclusion that the programs should not specifically focus on regions of higher or lower SES. Instead, the programs should be targeted according to the specific needs in the districts. Cultural-specific interventions could make use of cultural diversity in districts of lower SES. Moreover, higher SES districts could improve prevention of stigma by providing specific information on controllability of epilepsy and its comorbidities. School-based interventions should also cover these aspects in order to educate children, adolescents and their families early.

Our results further suggest that psychotherapy should regularly include the possible stigma of epilepsy. We found moderate to high correlations of depressive symptoms and anxiety symptoms with stigma (Table 2), suggesting that PWE seeking therapy due to these comorbidities may especially suffer from stigma. Therapists should create settings to allow for correcting relationship experiences. Therefore, information about epilepsy should be provided in psychotherapists' training to reduce their possible epilepsy-related restraints and stereotypes.

### Limitations, generalizability and further research

Due to the cross-sectional nature of our study, we cannot draw conclusions about the directions of the observed relationships. Longitudinal and qualitative research is needed to gain further insight in possible causal mechanisms. Moreover, we only focused on the patients' perception of stigma, i.e., rather subjective views of “felt stigma.” In addition to that, the concept of “enacted stigma” refers to actual episodes of discrimination, e.g., bullying due to epilepsy, representing a more objective perspective. For a comprehensive view on stigma and its everyday life consequences, both aspects should be taken into account in further studies. Our sample consists of patients from 2018 to 2021 whereas the latest version of the measures for structural SES, the social indices of Berlin, was published in 2013. However, the SIs of different periods (e.g., 2008 and 2013) are usually highly correlated (37, 52), so that if any, only a small bias due to changes of the social structure of Berlin in our results is expected. Moreover, our in-patient sample may have suffered from particularly severe epilepsies, and to draw conclusions about a more representative epilepsy population, outpatient settings should be examined as well. Finally, comparisons with other cities and countries are needed to examine whether our results were specific to Berlin and/ or Germany.

Moreover, a possible limitation of our study is that we did not include a control group. Additional research is needed to test whether our findings are generalizable to other chronic health conditions. For example, HIV-related or mental health stigma also showed associations with the structural SES in previous studies (16, 43). However, whether stigma against other conditions also differs even according to different regional levels within one city, needs to be proven. Directly comparing groups of different chronic medical illnesses with respect to the relations between SES and stigma may give additional insight in underlying mechanisms and treatment needs. Prevention and intervention programs may greatly benefit from the corresponding findings.

Stigma may play an important role for pathological social stress in urban populations. Our findings suggest that structural socioeconomic conditions of the living environment should be considered within this framework. However, our non-linear relationships indicate that at least some indicators of lower structural SES may not cause higher stigma levels. These findings may be particularly important within the interdisciplinary field of neurourbanism. Further research is needed to disentangle the mechanisms from an interdisciplinary perspective. For instance, instead of using a composite measure of SES, studies could investigate specific aspects of the residence, e.g., average household income rates, educational qualifications, cultural diversity and also the nature outdoor environment and green space (22, 43, 53). This may help to identify possible resilience factors in areas with lower structural SES.

## Conclusion

We found that the social structure of the residence was linked to perceived stigma in patients with epilepsy in Berlin. Interventions to reduce and prevent epilepsy-related stigma already exist but regarding their efficacy, outcomes are mixed (51). These patient-based and public interventions should take into account that perceived stigma varies according to the social environment and may, therefore, be improved taking into account different regional needs. All in all, our findings regarding epilepsy-related stigma may be transferred to stigma against other health conditions.

## Data availability statement

The raw data supporting the conclusions of this article will be made available by the authors, without undue reservation.

## Ethics statement

The studies involving human participants were reviewed and approved by Institutional Review Board of Charité–Universitätsmedizin Berlin. The patients/participants provided their written informed consent to participate in this study.

## Author contributions

LH collected and analyzed the data, together with MH she wrote the manuscript. S-UK and JB contributed to data collection and reviewed the manuscript. All authors contributed to the article and approved the submitted version.

## Funding

This study was supported by a grant (no number available) from the Epilepsy Academy Berlin-Bethel to LH. The funding source was not involved in the design of this study, the writing of this report and in the decision to submit the manuscript for publication.

## Conflict of interest

Author MH received speaker's honoraria and/or consultancy fees from Angelini, Bial, Desitin, Eisai, GW Pharmaceuticals, Neuraxpharm, UCB, and Zogenix. The remaining authors declare that the research was conducted in the absence of any commercial or financial relationships that could be construed as a potential conflict of interest.

## Publisher's note

All claims expressed in this article are solely those of the authors and do not necessarily represent those of their affiliated organizations, or those of the publisher, the editors and the reviewers. Any product that may be evaluated in this article, or claim that may be made by its manufacturer, is not guaranteed or endorsed by the publisher.
