# Supplementary material for: Perceived epilepsy-related stigma is linked to the socioeconomic status of the residence
Source: Front Public Health. 2022 Aug 26;10:952585. doi: 10.3389/fpubh.2022.952585 (PMC9459334; doi:10.3389/fpubh.2022.952585)
Supplement: Supplementary file 1 [file Table_1.DOCX]

Supplementary Material

# Translation of the ‘stigma’ scale

English translation of the subscale ‘stigma’ of an adapted version of the German Performance, socio-demographic aspects, subjective evaluation (PESOS) questionnaire

What do you think how other people react to you?

1. Would you have earned more appreciation if you had not have epilepsy?

2. Do you have more problems than other people with finding friends due to the epilepsy?

3. Are others withdrawing from you due to your epilepsy?

4. Are you viewed as less intelligent by others due to your epilepsy?

5. Are other people bewildered by your epilepsy?

6. Are other people are talking about you due to your epilepsy?

All questions are answered on a five-point scale with labels ‘very strong’, ‘strong’, ‘somewhat’, ‘a little’, ‘not at all’. Please note that this translation into English is not validated and was created solely for displaying the items to the international readership of the manuscript.
